# Antileukemic Potential of *Momordica charantia* Seed Extracts on Human Myeloid Leukemic HL60 Cells

**DOI:** 10.1155/2012/732404

**Published:** 2012-05-13

**Authors:** Ramani Soundararajan, Punit Prabha, Umesh Rai, Aparna Dixit

**Affiliations:** ^1^Gene Regulation Laboratory, School of Biotechnology, Jawaharlal Nehru University, New Delhi 110 067, India; ^2^Department of Zoology, University of Delhi, New Delhi 110 007, India

## Abstract

*Momordica charantia* (bitter gourd) has been used in the traditional system of medicine for the treatment of various diseases. Anticancer activity of *M. charantia* extracts has been demonstrated by numerous *in vitro* and *in vivo* studies. In the present study, we investigated the differentiation inducing potential of fractionated *M. charantia* seed extracts in human myeloid HL60 cells. We found that the HL60 cells treated with the fractionated seed extracts differentiated into granulocytic lineage as characterized by NBT staining, CD11b expression, and specific esterase activity. The differentiation inducing principle was found to be heat-stable, and organic in nature. The differentiation was accompanied by a downregulation of *c-myc* transcript, indicating the involvement of *c-myc* pathway, at least in part, in differentiation. Taken together these results indicate that fractionated extracts of *M. charantia* seeds possess differentiation inducing activity and therefore can be evaluated for their potential use in differentiation therapy for leukemia in combination with other inducers of differentiation.

## 1. Introduction

Acute myeloid leukemia (AML) is a complex disease, characterized by abnormal differentiation and unlimited cellular proliferation that develops due to the accumulation of genetic and epigenetic alterations. Current treatments including chemotherapeutic drugs, radiation, and stem cell transplantation are associated with incomplete remission and side effects [1]. Therefore, there is a need to discover novel anticancer drugs that are effective and have minimal side-effects associated with the treatment. In traditional system of medicine and folklores, plant-based formulations with potential anticancer properties have been described [2, 3]. Investigating dietary compounds with potential chemopreventive and anticancer activity has provided important leads for the development of clinically relevant anticancer drugs [3, 4] However, systematic scientific studies have been lacking to understand the mechanism (s) of their anticancer activity.

In the present study, we have focused our attention on a tropical plant, *Momordica charantia* L, belonging to Cucurbitaceae family that has been used in the traditional health care world over and the ethnobotanical use of this medicinal plant is well documented [5, 6]. *M. charantia* has been reported to possess a number of diverse medicinal properties such as antimicrobial, antidiabetic, antifertility and abortifacient activity [7–10]. Antigrowth properties of fractionated *M. charantia* whole plant extracts were first reported by West et al. [11]. Subsequently, a number of growth inhibitors have been isolated from *M. charantia* seeds and its antiproliferative activity has been demonstrated in a variety of tumor cell lines [8, 12–15]. *M. charantia* fruit extract and its components have also been shown to be cytotoxic to leukemic lymphocytes and induce antitumor activity *in vivo* [8, 16, 17].

While antiproliferative and antitumor activity of *M. charantia* has been demonstrated, its differentiation inducing potential in leukemic cell lines has not been investigated. We have used human myeloid leukemic HL60 cells as an *in vitro* system to test the differentiation inducing potential of *M. charantia* and to elucidate the molecular mechanisms involved in the differentiation process. HL60 cells have been used for screening of compounds for their antileukemic potential and to elucidate the molecular mechanisms involved in the differentiation process. Different compounds have been reported to induce HL60 cells towards granulocytic, macrophagic, and monocytic lineage [18–21]. In the present study, we report for the first time that the fractionated *M. charantia* seed extract induced differentiation of HL60 to granulocytes and thus has potential to be developed as a therapeutic supplement.

## 2. Material and Methods

### 2.1. Cells and Reagents

The human promyelocytic leukemia HL60 cells (ATCC#CCL240) were maintained in 1 × RPMI 1640 medium supplemented with fetal calf serum (FCS, 20%), penicillin (100 U/mL), and streptomycin (100 *μ*g/mL) and 2 mM L-glutamine in a 5% CO_2_ humidified atmosphere. The chemicals required for cell culture were procured from Biological Industries, Israel. Other chemicals were from standard commercial suppliers.

### 2.2. Extraction and Partial Purification of *M. charantia* Seed Extract

Seeds of* M. charantia L.* (Pusa Vishesh variety, developed, verified, and released by Indian Agriculture Research Institute (IARI), Pusa, New Delhi [22]) were obtained from IARI, Pusa, New Delhi. Decorticated seeds (100 g) were extracted in 75% ethanol containing 0.2 N HCl and 1 mM PMSF. The crude extract was centrifuged at 20,000 ×g for 1 hr at 4°C. The supernatant was evaporated to 1/4 volume under reduced pressure followed by neutralization using NH_4_OH. Differential fractionation of the extract (Mc-1, dry weight ~16.5 g) thus obtained using 0.05 M (NH_4_)_2_CO_3_ followed by centrifugation at 20,000 ×g for 45 min at 4°C resulted in the pellet (Mc-2, dry weight ~2.3 g) and supernatant (Mc-3, dry weight ~14.2 g) fractions. All the fractions were evaporated and resuspended in Milli-Q water prior to use. The fraction Mc-2 was further fractionated using acetone extraction (Mc-2Ac, dry weight ~400 mg). Protein was estimated using Lowry's method [23].

### 2.3. Cell Viability and Cell Proliferation Assay

Cell viability of HL60 cells upon treatment with *M. charantia* fractions, namely, Mc-1, Mc-2, Mc-3, and Mc-2Ac was assayed using MTT essentially as described by Yedjou et al. [24]. HL60 cells were seeded in 96-well plate at an initial density of 10^5^ cells/mL. Different concentrations of *M. charantia *test fractions (diluted in the medium) were added to the cells. The blank sample contained medium only. The cells were placed in humidified 5% CO_2_ incubator at 37°C. After 72 h of incubation, the cells were centrifuged at 800 rpm for 5 min and the supernatant culture medium carefully aspirated. The cells were given 2 washes with PBS, followed by addition of 200 *μ*L fresh medium containing MTT (0.5 mg/mL). The cells were further incubated at 37°C for 2 h, followed by centrifugation at 800 rpm for 5 min. The medium containing MTT was carefully removed followed by addition of 100 *μ*L DMSO to each well. The plate was incubated at room temperature with shaking for 10 min to solubilize formazan crystals. The absorbance at 570 nm was measured in a microplate reader (Tecan, Switzerland). The amount of color produced is directly proportional to the number of viable cells. All assays were done in triplicates for each concentration and cell viability was estimated by using means + SD values for each treatment. Cell viability was calculated using MTT absorbance of the control and treated cells: % survival = (mean value treated sample/mean value of untreated sample) ∗ 100.

Effect of *M. charantia* fractions on cell proliferation rate was determined by viable cell count using a hemocytometer as described by Lee et al. [20]. Briefly, HL60 cells (10^5^ cells/mL) were plated in a 24-well plate and incubated with different fractions of *M. charantia* at a final concentration of 20 *μ*g/mL for 5 days. Viable cell counts were determined on each day post treatment using trypan blue dye exclusion assay using a light microscope (Leica, Germany).

### 2.4. Differentiation of HL60 Cells

HL60 cells (2 × 10^5^/mL) were incubated with the test fractions at the indicated concentrations for different time intervals. Cell differentiation was assayed by NBT (Nitroblue tetrazolium) reduction, Wright-Giemsa staining, specific and nonspecific esterase activities, and by FACS analysis.

### 2.5. Nitroblue Tetrazolium (NBT) Reduction

NBT staining was used to assay for HL60 cell differentiation essentially as described earlier [25]. Approximately 200 cells in triplicates were scored under a light microscope (Leica, Germany) for each test sample. Extent of differentiation is expressed as number of % NBT positive cells. The data corresponding to the number of total NBT positive cells out of the total cells counted for different experiments are given in Table  1 in supplementary material available online at doi: 10.1155/2012/732404. The data were analyzed using one-way ANOVA and Tukey post hoc test to compare the difference between the treated and the untreated control. An alpha value of <0.05 was considered to be significant. 

### 2.6. Wright-Giemsa Staining

Morphological changes in the HL60 cells induced by the test samples were evaluated using Wright-Giemsa staining as per the manufacturer's instructions (Sigma-Aldrich, USA). Differential cell count was performed under a light microscope (Leica, Germany) from at least 3 independent glass slides for each experimental time point in triplicates.

### 2.7. Specific and Nonspecific Esterase Activity

Specific and nonspecific esterase activities in the HL60 cell lysates of the control and treated cells were estimated using cytochemical kits as per manufacturer's instruction (Sigma 91-A and 91-C, Sigma-Aldrich, USA). The presence of granulation (reddish and blackish granulation indicating the specific and nonspecific esterase, respectively) indicated the areas of enzyme activity, which was scored in a scale of 0 to +4, 0 indicating no staining, hence no enzyme activity and +4 indicating brilliant staining, and hence maximal enzyme activity. 

### 2.8. Analysis for CD11b Expression

Control and treated HL60 cells (5 × 10^5^ cells) washed with PBS containing 1% FCS and 0.01% sodium azide were incubated for 30 min in fetal calf serum at 4°C. Subsequently, FITC-conjugated anti-human CD11b antibody (1 *μ*g/mL, eBiosciences, USA) was added to the cells and incubated at 25°C for 45 min followed by washing with PBS. The cells were then fixed in 1% paraformaldehyde and analyzed on Becton Dickinson Flow cytometer. Isotypic control (Rat IgG2b) was also used to check for nonspecific binding. The results were plotted using WinMdi software (Flow Core Facility, Scripps Research Institute; http://facs.scripps.edu/software.html). 

### 2.9. Northern Blot Analysis of c-myc Transcript

HL60 cells (2 × 10^5^ cells/mL) were treated with fractions Mc-2 and Mc-2Ac (20 *μ*g/mL each). Total RNA from the control and treated cells was isolated on day 4 of the treatment using TRIzol reagent (Life Technologies, USA) as per the manufacturer's instructions. Briefly, total RNA (10 *μ*g each) isolated from the control and treated HL60 cells resolved on 1% formaldehyde agarose gel was transferred onto nitrocellulose membrane and subjected to Northern blotting using *α*-P^32^-dCTP-labeled *c-myc *gene specific probe [26] essentially as described earlier [27].

## 3. Results

### 3.1. Acid Ethanol Extractable Fractions of *M. charantia* Seeds Induce Differentiation of HL60 Cells

**Figure 1:**
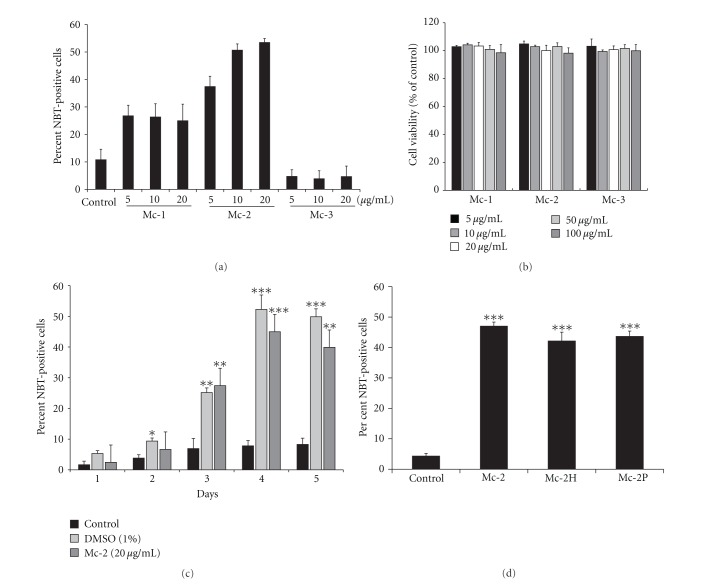
(a) Effects of different fractions of *M. charantia* seeds on HL60 cells differentiation. HL60 cells treated with varying concentrations of different fractions were scored for the percentage of NBT positive cells on day 5. Mc-1-(5–10 *μ*g/mL) and Mc-2-(5–20 *μ*g/mL) treated cells showed a significant increase in NBT staining compared to untreated control. (b) Effect of different fractions of *M. charantia* seeds on HL-60 cell viability. HL60 cells (10^5^ cells/mL) were cultured in 96-well plates and incubated with the indicated concentrations of fractions Mc-1, Mc-2, and Mc-3, for 72 h. The MTT assay was used to assess cell viability. The optical density value of the control was regarded as 100%. Data represent the mean ± SD of three independent experiments. (c) Time dependence of Mc-2 induced HL60 cells differentiation. Control, and Mc-2 (20 *μ*g/mL) or DMSO-(included as a positive control) treated HL60 cells were scored for percentage of differentiation by NBT staining from day 1 to 5. (d) Effect of heat-inactivated Mc-2 (Mc-2H) and *S. griseus* protease-treated (Sigma Aldrich, USA) Mc-2 (Mc-2P) on HL60 cells differentiation. HL60 cells treated with indicated fractions (20 *μ*g/mL each) were scored for the NBT positive cells on day 5. All the three fractions resulted in significantly increased NBT staining, relative to untreated control. The data in all the panels represent mean ± SD of three independent experiments performed in triplicates. *P < 0.05, **P < 0.01, and ***P < 0.001 relative to untreated control.

 Analysis of differentiation in HL60 cells treated with fraction Mc-1 revealed that it was capable of inducing differentiation as established by an increase in number of NBT positive cells. Maximum differentiation (~30%) was observed at 10 *μ*g/mL of Mc-1 (Figure 1(a)). However, treatment with higher concentrations of Mc-1 (20 *μ*g/mL) resulted only in 22–25% differentiation. Further fractionation of Mc-1 into fractions Mc-2 and Mc-3 resulted in the enrichment of the differentiation-inducing principle(s) in fraction Mc-2. Approximately 50% cells stained positive with Mc-2 (10 *μ*g/mL) with no further increase in differentiation at higher concentration (20 *μ*g/mL). The fraction Mc-3 showed minimal differentiation (Figure 1(a)).

In order to assess if the observed increase in NBT positive cells is due to death of immature cells caused by the test fractions, cytotoxicity analysis was carried out with the fractions Mc-1, Mc-2, and Mc-3 of *M. charantia. *No effect on the cell viability was observed with any of the fractions upto 100 *μ*g/mL (the maximum concentration tested; Figure 1(b)). These data indicate that the three fractions were noncytotoxic even at 5× concentrations than those used for differentiation studies (Figure 1(a)). Similar results were also obtained using Alamar blue reduction assay for cell survival. Percentage reduction of Alamar blue in HL60 cells treated with the three factions was comparable to the control cells confirming that the *M. charantia* fractions were noncytotoxic (data not shown).

### 3.2. Time Course of Induction of Differentiation

To investigate the time kinetics of the differentiation induced by fraction Mc-2, the HL60 cells were treated with Mc-2 at a final concentration of 20 *μ*g/mL and scored for NBT-positive cells from day 1 to day 5 of the treatment. As evident from Figure 1(c), differentiation was observed on day 3, which increased further on day 4 after which no significant change in the percentage of differentiated cells was observed.

### 3.3. The Differentiation Inducing Component(s) Present in Fraction Mc-2 Are Nonproteinaceous and Heat-Stable

In order to establish the nature of the component(s) responsible for inducing differentiation, fraction Mc-2 was heat-treated at 70°C (Mc-2H) or protease-treated (Mc-2P) for 30 min prior to its use in differentiation. No significant loss in the differentiation inducing ability was observed by these treatments when compared to fraction Mc-2 (Figure 1(d)) suggesting that the differentiation inducing principle(s) are nonproteinaceous in nature.

**Figure 2:**
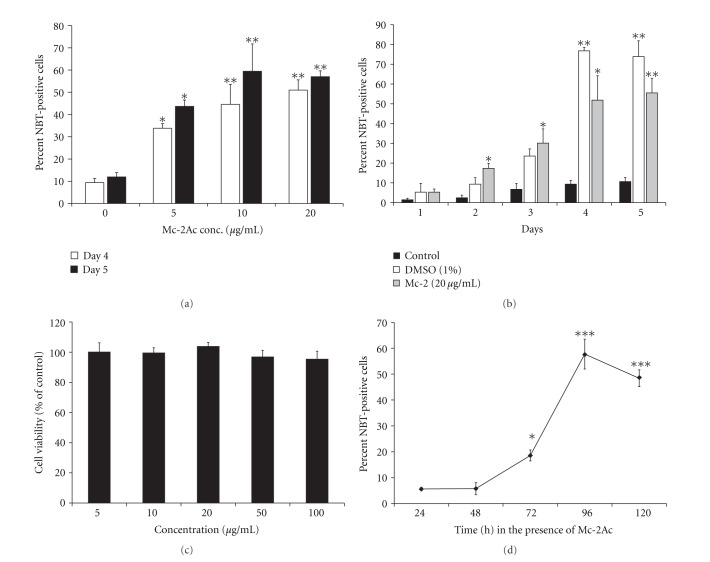
(a) Effect of acetone extract of Mc-2 (Mc-2Ac) on HL60 cells differentiation. Mc-2Ac-treated (5–20 *μ*g/mL) cells showed a significant increase in NBT positive cells on day 4 and day 5, respectively, compared to untreated cells. (b) Time-dependence of Mc-2Ac-induced HL60 cells differentiation. Mc-2Ac-treated HL60 cells were scored for NBT-positive cells from day 1 to 5. DMSO was included as a positive control. (c) Effect of fraction Mc-2Ac on HL60 cells viability. HL60 cells (10^5^/mL) cultured in a 96-well plate were treated with the indicated concentrations of fraction Mc-2Ac for 72 h. Cell viability was assessed by MTT assay. The optical density values of untreated control cells were taken as 100%. The data represent the mean + SD of three independent experiments. (d) Minimum time required for Mc-2Ac to induce HL60 differentiation. HL60 cells cultured in the presence of Mc-2Ac (20 *μ*g/mL) for the indicated time, followed by growth in the medium without Mc-2Ac, were scored for NBT-positive cells on day 5. Data in all the panels represent the mean ± S.D of 3 independent experiments performed in triplicates. *P < 0.05, **P < 0.01, and ***P < 0.001 relative to untreated cells.

Further since the principle(s) were found to be nonproteinaceous in nature, different concentrations of acetone extractable component(s) of fraction Mc-2 (designated as Mc-2Ac) were tested for its differentiation inducing potential. The fraction Mc-2Ac induced differentiation in a concentration- and time-dependent manner (Figures 2(a) and 2(b)). The fraction Mc-2Ac showed higher differentiation inducing potential. As evident from the data, fraction Mc-2 showed between 50 and 53% differentiation at 10 and 20 *μ*g/mL, whereas fraction Mc-2Ac was able to bring about 59% differentiation even at 10 *μ*g/mL concentration (cf. Figures 1(a) and 2(a)). Like the parent fraction Mc-2, fraction Mc-2Ac also did not show any cytotoxic effect on HL60 cells even at 5× concentration than that used for differentiation (Figure 2(c)).

### 3.4. Minimum Time Required for Fraction Mc-2Ac to Trigger Differentiation

To determine the minimum time required for the differentiation to be triggered by fraction Mc-2Ac, the cells were grown in the medium containing fraction Mc-2Ac (20 *μ*g/mL) for different time period followed by their growth in normal medium. Very low percentage of cells was NBT-positive when the cells were grown in the medium containing Mc-2Ac up to 48 h (Figure 2(d)). A significant increase in the NBT-positive cells was recorded when the cells were grown in the medium containing Mc-2Ac for 72 h. Further increase in the time for which the fraction Mc-2Ac was present in the medium resulted in further increase in the NBT-positive cells. The data suggest that fraction Mc-2Ac is required to be present in the medium for at least 72 h to trigger differentiation. The cells show maximum differentiation (~55–59%) when the fraction Mc-2Ac remained present in the medium for 96 h.

### 3.5. Effect of *M. charantia* Seed Fractions on HL60 Cells Proliferation

**Figure 3:**
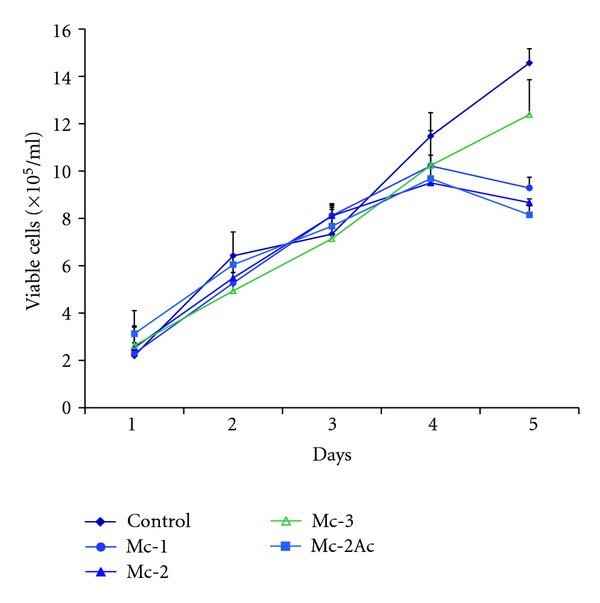
The effect of different fractions of *M. charantia* seed extract on the growth of HL60 cells. Cells were treated with different concentrations of fractions Mc-1, Mc-2, Mc-3 and Mc-2Ac (each at 20 *μ*g/mL concentration) for 5 days. Viable cell count was determined at different posttreatment intervals. The data represent means ± SD of three parallel experiments.

Since many other inducers of differentiation are known to cause both differentiation and inhibit proliferation [28–30], effect of different fractions of *M. charantia* was also evaluated on proliferation of HL60 cells. As evident from Figure 3, no change in rate of proliferation was noted till day 3 of the treatment, after which a slight decline in the cell count was observed on day 4 and day 5 in the treated samples, when compared to the untreated control.

### 3.6. CD11b Expression in Mc-2Ac-Treated HL60 Cells Confirms Differentiation

**Figure 4:**
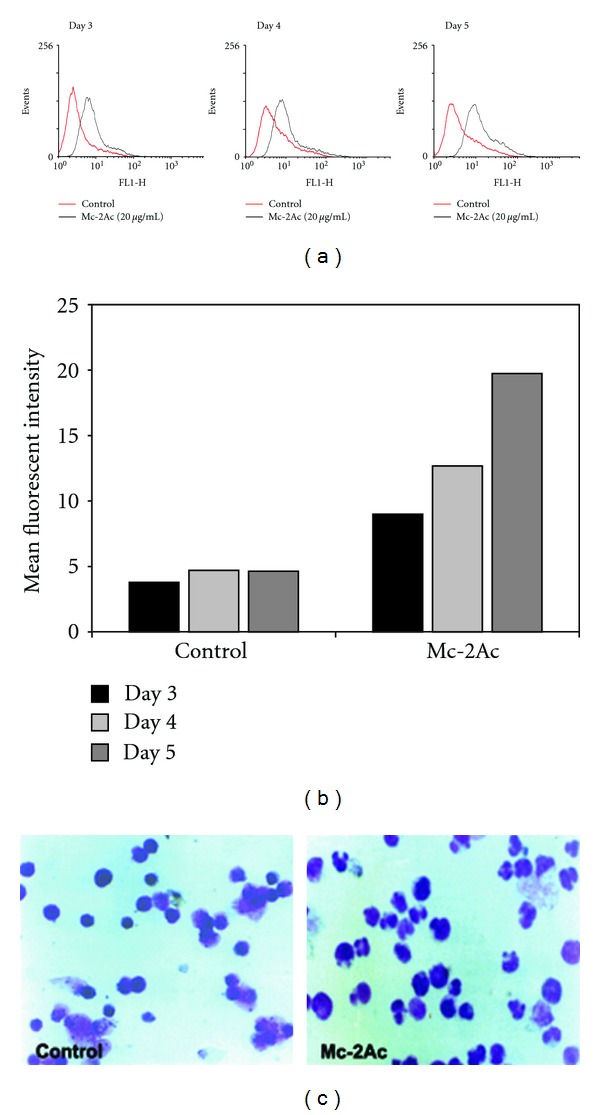
(a) Analysis of CD11b expression. Untreated control and Mc-2Ac- (20 *μ*g/mL) treated HL60 cells were analyzed for CD11b expressing population by FACScan. (b) Mean fluorescent intensity of CD11b expression in untreated control, and Mc-2Ac- (20 *μ*g/mL) treated cells from day 3 to day 5. (c) Wright-Giemsa-stained untreated control and Mc-2Ac-treated HL60 cells on day 5 of the treatment. Differentiated cells with distinct granulocytic features, that is, multilobular nuclei can be seen in Mc-2Ac-treated cells (40×).

Differentiation of HL60 cells treated with Mc-2Ac was further confirmed by analysis of expression of CD11b, a marker of differentiation. An increase in the presence of CD11b positive cells could be detected in the Mc-2Ac-treated HL60 cells from day 3 to day 5, evident by the observed increase in the mean fluorescent intensity, when compared to untreated control (Figures 4(a) and 4(b)). Negligible staining was seen with isotype control and incubation of cells with isotype control Mab did not change cd11b expression (data not shown). The percentage increase in the expression of CD11b in Mc-2Ac-treated cells correlated well with the number of NBT-positive cells from day 3 to day 5. 

### 3.7. The Acetone Extractable Fraction (Mc-2Ac) Differentiates HL60 Cells into Granulocytes

HL60 cells are predominantly promyelocytic in origin and can be induced to differentiate into granulocytic or monocytic lineages by different inducers. Wright-Giemsa staining of the fractions Mc-2- and Mc-2Ac-treated HL60 cells reveals the presence of HL60 cells of granulocytic lineage. Presence of segmented and banded neutrophils in Mc-2Ac-treated cells is evident from Figure 4(c).

Lineage of differentiation was further confirmed by determination of the specific/nonspecific esterase activity in HL60 cells. The HL60 cells treated with either Mc-2 or Mc-2Ac were found to be positive for specific esterase activity (+2 to +3) and negative (0) for nonspecific esterase activity confirming to the granulocytic differentiation of the HL60 cells by *M. charantia* seed fractions.

### 3.8. Effect of Fraction Mc-2Ac on *c-myc* Expression in HL60 Cells

**Figure 5:**
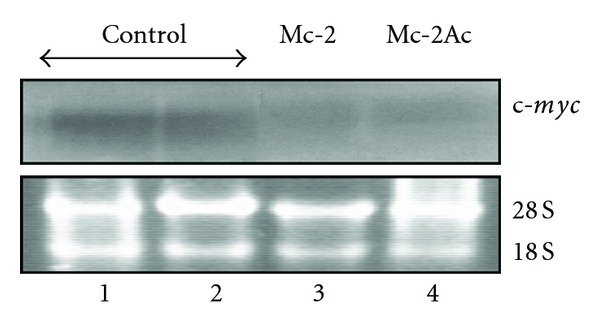
Northern blot analysis of c-*myc *expression in the control (lanes 1 and 2), Mc-2-(lane 3), and Mc-2A-(lane 4) treated HL60 cells. Top panel shows the 2.1 Kb c-myc transcripts. An identical gel stained with ethidium bromide is included as loading control (bottom panel).

Since *c-myc* expression has been reported to be downregulated in leukemic cells that were induced to differentiate [31–33], *c-myc* transcript levels were measured in HL60 cells treated with fractions Mc-2 and Mc-2Ac on day 4 after treatment. Northern blot analysis performed with total RNA isolated from the control and treated cells revealed a significant decrease in the levels of *c-myc* transcript (2.1 Kb) in the Mc-2Ac-treated HL60 cells when compared to untreated control (Figure 5). These data suggest that the differentiation induction by *M. charantia* seed fraction may, in part, involve *c-myc* pathway.

## 4. Discussion

In the present study, we for the first time demonstrate the differentiation induction potential of the *M. charantia* seeds in HL60 cells. It is clearly demonstrated that fraction Mc-1 and its subsequent fractions Mc-2 and Mc-2Ac are able to differentiate HL60 cells. As Mc-1 is crude extract, a decrease in the differentiated cells in Mc-1-treated cells at higher concentration (i.e., 20 *μ*g/mL) in comparison with that observed with 10 *μ*g/mL could possibly be due to the presence of some compounds that could inhibit differentiation and which gain their inhibitory effect when concentration is increased to 20 *μ*g/mL. Upon further fractionation into Mc-2 and Mc-3, these inhibitors are fractionated away and that is why no decrease in differentiation is seen in fractions Mc-2 at 20 *μ*g/mL.

The test fractions of *M. charantia* capable of inducing differentiation did not exhibit any cytotoxic effect on HL60 cells. These data suggest that the increase in NBT-positive differentiated cells upon treatment is indeed due to differentiation brought about by the *M. charnatia* fractions and not caused by cell death of immature undifferentiated cells. Our results are in agreement with the reports put forth by Sharma et al., who also did not observe any cytotoxic effect of ethanolic extract of *M. charantia* on a number of cancer cells [34].

Unlike many other plant-derived compounds which induce the HL60 cells towards monocytic or macrophagic pathway [35, 36], fractionated *M. charantia* extracts induced HL60 cells into granulocytic pathway, evident by the morphological characteristics of granulocytic lineage, presence of specific esterase activity, and expression of CD11b in the Mc-2- and Mc-2Ac-treated HL60 cells. Granulocytic differentiation of HL60 cells by fractionated *M. charantia* seed extract is comparable to that observed with retinoic acid [25].

Induction of differentiation of HL60 cells by fractionated *M. charantia* seed extracts in a time- and concentration-dependent manner supports the “all or none” response similar to that of DMSO and sodium butyrate [37] as opposed to the graded response seen by another inducer of HL60 differentiation such as retinoic acid [25]. It is known that different inducers require different lengthes of time to trigger differentiation in different cell types [38–40]. During this period, the cells become committed to differentiation and this has been proposed to be a rate limiting step for the cells to synthesize cellular constituents above a threshold value [41]. While difluoromethylornithine (DFMO) is reported to trigger differentiation of MEL cells when present in the medium for only 24 h, vitamin D3 is required to be present for 48 h in the medium to induce differentiation of MEL cells [40]. On the other hand, vitamin D3 was able to induce differentiation of HL60 cells on day 1 itself [38]. In the present study, it was established that presence of Mc-2Ac for a minimum time of ~72 h is required to observe any significant differentiation, though some differentiation could be observed on day 2 also. Since the fraction Mc-2A did not show any cytotoxic effect even at 2.5× concentration that induced maximum differentiation, it is advantageous over other inducers such as DFMO or DMSO, that exhibit cytotoxic effect at higher concentration.

The nature of the differentiation inducing compound(s), as established by the retention of the differentiation inducing activity even after heat and protease treatment, is in line with other studies wherein a large proportion of the inducers of HL60 differentiation are nonproteinaceous in nature [35, 36]. The extent of differentiation induced with the Mc-2 and Mc-2Ac fractions is comparable with the differentiation induced by many other plant-derived compounds [42].

*M. charantia* seed fractions did not exhibit any antiproliferative activity. Till day 3, there was no change in the growth profile of the treated cells when compared to the untreated cells. Similar results have been obtained by induction of differentiation of human K562 cells with hemin [43]. A slight decline in the cell count in Mc-1, Mc-2, and Mc-2Ac fractions on day 4 and day 5 of the treatment is due to induction of differentiation as the differentiated cell population does not proliferate further. These data suggest that the test fractions do not possess antiproliferative activity. Although *M. charantia* extracts have been reported to have antitumor and antiproliferative activities ([13–16], for review please see [10, 44]), most of such activities have been attributed to a number of proteins identified as lectins—ribosome inactivating proteins such as alpha momorcharin, momordin [7, 15, 45], and MAP30 ([8, 46], for review, please see [10, 44]). Unlike these, the differentiation inducing fractions did not exhibit any antiproliferative activity and the differentiation inducing principle(s) was found to be nonproteinaceous in nature, suggesting that the differentiation inducing factors of *M. charantia* are different from its antitumor factors.

Since HL60 cells are induced to differentiate by retinoic acid, it was imperative to rule out the possibility that the differentiation by *M. charantia* extract is not due to retinoic acid present in the extracts, if any. For this purpose, differentiation inducing potential of the fraction Mc-1 was assessed in K562 cells, prototype human myeloid leukemia cell type that is resistant to retinoic acid induced differentiation [28, 47] by morphological examination as described earlier [48]. While treatment with positive inducer sodium butyrate resulted in upto 85–90% cells to be differentiated, fraction Mc-1 resulted in differentiation of ~40–45% cells (Ramani Soundararajan, Madhu Geeta, and Aparna Dixit, unpublished data). Vitamin A levels have been reported to be undetectable in methanolic extracts of *M. charantia *[49]. In the present study, seed extraction was performed with acid ethanol, and therefore, it was unlikely to have retinoic acid. Analysis of the test fractions Mc-1, Mc-2, Mc-3, and Mc-2A for vitamin A content by LC-MS-MS confirmed that these fractions did not contain vitamin A (data not shown). Thus, the ability of *M. charantia* seed extract to induce differentiation of vitamin A resistant K562 cells and absence of vitamin A in the test fractions of *M. charantia* suggest that the differentiation brought about by these fractions in the present study is not brought about by retinoic acid.

The transcription factor c-Myc plays a role in the control of cell proliferation and differentiation [50]. A significant decrease in the *c-myc* expression in the Mc-2- and Mc-2Ac-treated cells is in line with previous reports wherein a modulation in *c-myc* expression in HL60 cells induced to differentiate has been established [31, 32]. Murine erythroleukemic (MEL) cells induced to differentiate by DMSO also showed a biphasic change in the *c-myc *levels suggesting that the changes in *c-myc* may be important for irreversible commitment of MEL cells towards terminal erythroid lineage [33]. The reduced expression of *c-myc* can be related to the loss of proliferative potential of the differentiated cells [32]. A slight decrease in *c-myc* transcript in the Mc-2Ac-treated cells relates well with the decrease in number of cells from day 3 onwards, relating to the loss of proliferating potential of the differentiated cells as c-Myc has been shown to be functionally involved in DNA synthesis [51].

Thus, the present study for the first time demonstrates differentiation inducing activity of the fractionated *M. charantia* seed extracts which can be further assessed as inducer of differentiation for the treatment of APL, either alone or in combination with the suboptimal concentrations of other known inducers of differentiation. Studies are in progress to understand the mechanism by which the compounds present in fraction Mc-2Ac are inducing differentiation.

## Supplementary Material

Supplementary Table 1A: Effect of different fractions of *M. charantia* seeds on HL60 cells differentiation on day 5 of the treatment. HL60 cells treated with varying concentration (5-20 **µ**g/ml) of different fractions (Mc-1, Mc-2 and Mc-3) were scored for NBT positive cells on day 5. The data represent total number of cells, and total number of NBT positive cells in the respective samples scored in three wells (triplicates) for each experimental conditions.
Supplementary Table 1B: Time dependence of Mc-2 induced HL60 cells differentiation. HL60 cells were treated with Mc-2 (20 **µ**g/ml) and DMSO (1%) and scored for NBT staining from day 1 to 5. DMSO was included as a positive control. The data represent total number of cells, and total number of NBT positive cells in the respective samples scored in three wells (triplicates) for each experimental conditions.
Supplementary Table 1C: Effect of heat-inactivated Mc-2 (Mc-2H) and protease-treated Mc-2 (Mc-2P) on HL60 differentiation. HL60 cells treated with Mc-2H and Mc-2P (20 **µ**g/ml each) were scored for NBT positive cells. The data represent total number of cells, and total number of NBT positive cells in the respective samples scored in three wells (triplicates) for each experimental conditions.
Supplementary Table 1D: Effect of acetone extract of Mc-2 (Mc-2Ac) on HL60 differentiation. Mc-2Ac - treated (5-20 **µ**g/ml) were scored for NBT positive cells on day 4 and day 5, respectively. The data represent total number of cells, and total number of NBT positive cells in the respective samples scored in three wells (triplicates) for each experimental conditions.
Supplementary Table 1E: Time dependence of Mc-2Ac induced HL60 differentiation. Mc-2Ac-treated HL60 cells were scored for NBT-positive cells from day 1 to day 5. DMSO was included as a positive control. The data represent total number of cells, and total number of NBT positive cells in the respective samples scored in three wells (triplicates) for each experimental conditions.
Supplementary Table 1F: Minimum time required for Mc-2Ac to induce differentiation. HL60 cells cultured in the presence of Mc-2Ac for the indicated time, followed by growth in medium without Mc-2Ac were scored for NBT-positive cells on day 5. The data represent total number of cells, and total number of NBT positive cells in the respective samples scored in three wells (triplicates) for each experimental conditions.

## Abbreviations

DMSO: Dimethyl sulfoxide
DFMO: Difluoromethylornithine
PMSF: Phenylmethylsulfonyl fluoride
NBT: Nitroblue tetrazolium.
