# Supplementary material for: Antileukemic Potential of Momordica charantia Seed Extracts on Human Myeloid Leukemic HL60 Cells
Source: Evid Based Complement Alternat Med. 2012 May 13;2012:732404. doi: 10.1155/2012/732404 (PMC3359694; doi:10.1155/2012/732404)
Supplement: Supplementary file 1 — Supplementary Table 1A: Effect of different fractions of M. charantia seeds on HL60 cells differentiation on day 5 of the treatment. HL60 cells treated with varying concentration (5-20 µg/ml) of different fractions (Mc-1, Mc-2 and Mc-3) were scored for NBT positive cells on day 5. The data represent total number of cells, and total number of NBT positive cells in the respective samples scored in three wells (triplicates) for each experimental conditions. Supplementary Table 1B: Time dependence of Mc-2 induced HL60 cells differentiation. HL60 cells were treated with Mc-2 (20 µg/ml) and DMSO (1%) and scored for NBT staining from day 1 to 5. DMSO was included as a positive control. The data represent total number of cells, and total number of NBT positive cells in the respective samples scored in three wells (triplicates) for each experimental conditions. Supplementary Table 1C: Effect of heat-inactivated Mc-2 (Mc-2H) and protease-treated Mc-2 (Mc-2P) on HL60 differentiation. HL60 cells treated with Mc-2H and Mc-2P (20 µg/ml each) were scored for NBT positive cells. The data represent total number of cells, and total number of NBT positive cells in the respective samples scored in three wells (triplicates) for each experimental conditions. Supplementary Table 1D: Effect of acetone extract of Mc-2 (Mc-2Ac) on HL60 differentiation. Mc-2Ac - treated (5-20 µg/ml) were scored for NBT positive cells on day 4 and day 5, respectively. The data represent total number of cells, and total number of NBT positive cells in the respective samples scored in three wells (triplicates) for each experimental conditions. Supplementary Table 1E: Time dependence of Mc-2Ac induced HL60 differentiation. Mc-2Ac-treated HL60 cells were scored for NBT-positive cells from day 1 to day 5. DMSO was included as a positive control. The data represent total number of cells, and total number of NBT positive cells in the respective samples scored in three wells (triplicates) for each experimental conditi [file 732404.f1.pdf]

SUPPLEMENTARY TABLES 1A-1F CORRESPONDING TO FIGURES 1A,1C,1D,2A,2B AND 2D, RESPECTIVELY

Table 1A: Effect of different fractions of *M. charantia* seeds on HL60 cells differentiation on day 5 of the treatment.

**Treatment**	**Mc-1**			**Mc-2**			**Mc-3**		
**Conc. (µg/ml)**	**5**	**10**	**20**	**5**	**10**	**20**	**5**	**10**	**20**
Total number of cells counted	608	650	638	408	547	534	535	611	628
Number of NBT positive cells	162	169	158	154	280	285	25	26	28

DMSO (1%), a positive inducer resulted in 294 NBT positive cells out of 578 cells counted.

Table 1B: Time dependence of Mc-2 induced HL60 cells differentiation

Days after treatment	Control		Mc-2A (20 mg/ml)		DMSO (1%)	
	Total no. of Counted cells	No. of NBT positive cells	Total no. of Counted cells	No. of NBT positive cells	Total no. of Counted cells	No. of NBT positive cells
1	544	9	542	14	500	26
2	548	22	593	40	647	61
3	620	40	613	169	580	127
4	542	45	566	253	469	248
5	528	44	648	258	610	303

Table 1C: Effect of heat-inactivated Mc-2 (Mc-2H) and protease-treatedMc-2 (Mc-2P) on HL60 cells differentiation

Treatment	Control	Mc-2 (20 µg/ml)	Mc-2H(20 µg/ml)	Mc-2P (20 µg/ml)
Total no. of counted cells	631	634	590	554
No. of NBT positive cells	27	298	247	240

Table 1D: Effect of acetone extract of Mc-2 (Mc-2Ac) on HL60 cells differentiation

Treatment	Final Conc. (µg/ml)	Day 4		Day5	
		Total No.of counted cells	No. of NBT Positive cells	Total No.of counted cells	No. of NBT Positive cells
Control	-	444	40	587	72
Mc-2Ac	5	586	197	598	262
	10	801	340	542	324
	20	691	353	706	403

**Table 1E: Time dependence of Mc-2A induced HL60 cells differentiation**						
Days after treatment	Control		Mc-2A (20 mg/ml)		DMSO (1%)	
	Total no. of Counted cells	No. of NBT positive cells	Total no. of Counted cells	No. of NBT positive cells	Total no. of Counted cells	No. of NBT positive cells
1	579	9	626	34	615	35
2	628	15	583	100	612	57
3	652	45	616	195	636	147
4	625	59	669	339	567	435
5	590	62	718	386	578	424

**Table 1F : Minimum time required for Mc-2Ac to induce differentiation**					
Hours in the presence of Mc-2Ac (20 µg/ml)	24	48	72	96	120
Total number of cells counted	608	650	638	408	547
Number of NBT positive cells	162	169	158	154	280
